# Protective effect of naringenin on cadmium chloride-induced renal injury via alleviating oxidative stress, endoplasmic reticulum stress, and autophagy in chickens

**DOI:** 10.3389/fphar.2024.1440877

**Published:** 2024-07-12

**Authors:** Yaning Shi, Zhixin Gao, Bing Xu, Junbing Mao, Yue Wang, Zongping Liu, Jicang Wang

**Affiliations:** ^1^ College of Animal Science and Technology, Henan University of Science and Technology, Luoyang, China; ^2^ College of Veterinary Medicine, Yangzhou University, Yangzhou, China

**Keywords:** cadmium chloride, naringenin, oxidative stress, endoplasmic reticulum stress, autophagy

## Abstract

Cadmium (Cd) is a highly hazardous toxic substance that can cause serious harm to animals. Previous studies have indicated that cadmium chloride (CdCl_2_) can damage organs, such as the liver, ovaries, and testicles. Naringenin (Nar) represents a flavonoid with various properties that promote the alleviation of Cd-induced damage. In this experiment, 60 chickens were divided into the control group, 150 mg/kg CdCl_2_ treatment group, 250 mg/kg Nar treatment group, and 150 mg/kg CdCl_2_ + 250 mg/kg Nar co-treatment group, which were treated for 8 weeks. Kidney tissues samples were collected to investigate kidney function, including oxidative stress (OS), endoplasmic reticulum (ER) stress, and autophagy activity. Experimental results showed the decreased weight of chickens and increased relative weight of their kidneys after CdCl_2_ treatment. The increase in NAG, BUN, Cr, and UA activities, as well as the increase in MDA and GSH contents, and the decrease activities of T-AOC, SOD, and CAT in the kidney, manifested renal injury by OS in the chickens. TUNEL staining revealed that CdCl_2_ induced apoptosis in renal cells. CdCl_2_ upregulates the mRNA and protein expression levels of GRP78, PERK, eIF2α, ATF4, ATF6, CHOP, and LC3, and inhibited the mRNA and protein expression levels of P62 proteins, which leads to ER stress and autophagy. The CdCl_2_ + Nar co-treatment group exhibited alleviated CdCl_2_-induced kidney injury, OS, ER stress, and autophagy. Research has demonstrated that Nar reduces CdCl_2_-induced kidney injury through alleviation of OS, ER stress, and autophagy.

## 1 Introduction

Cadmium (Cd) is a prevalent environmental pollutant. The sources of Cd are very diverse, including industrial and agricultural activities, such as lead and zinc mines, nonferrous metal smelting, battery production, electroplating, plastics manufacturing, and pesticide production (Al Olayan et al., 2020; Charkiewicz et al., 2023). Human activities are also the main sources of Cd, such as copper and nickel smelting, burning of fossil fuels, phosphate-fertilizer production, and other activities that may lead to Cd release (Nna et al., 2017). The main exposure route of Cd is through respiratory inhalation, followed by food and water intake, with skin absorption being the least common. Once Cd enters the body, it is transported through the bloodstream to various organs, where it accumulates in the kidneys and liver. Its elimination from the body is relatively slow (Genchi et al., 2020). Cd adversely affects human and animal health, and it can damage the liver and kidney tissues of animals (Ali et al., 2022). Cd and its compounds are also recognized as human carcinogens. Their effects can lead to various types of cancer, such as breast cancer, gastrointestinal tumor, lung cancer, and prostate cancer (Huff et al., 2007).

The kidneys are a vital component of the urinary system, playing a crucial role in filtering impurities from the blood and regulating fluid balance (Osborn et al., 2021). Research indicated that the kidneys are one of the main organs affected by Cd exposure (Chen et al., 2019). Poultry is an important source of animal protein in the human diet. The European Commission has set a maximum safe level of Cd in poultry kidneys at 1.0 mg/kg (Commission Regulation, 2024). However, environmental factors such as feed, soil, and water contaminated with high concentrations of Cd may pose a threat to poultry health (Aljohani, 2023). Cd can also be gradually absorbed by the human body through the consumption of Cd-contaminated plants and animals, thereby posing a potential health hazard. Therefore, reducing the impact of Cd exposure on poultry, particularly chickens, has great significance.

Cd-induced oxidative stress (OS) serves as the primary cause of kidney injury (Ge et al., 2019). OS is an imbalance in the body’s oxidative and antioxidant systems caused by harmful stimuli or the removal of senescent cells from the body. Antioxidant enzymes include superoxide dismutase (SOD), catalase (CAT), glutathione peroxidase (GPx), and non-enzymatic compounds such as glutathione (GSH). They are endogenous antioxidants in the body and play a crucial role in maintaining redox homeostasis in body cells. Malondialdehyde (MDA) is the product of lipid peroxidation. By measuring MDA content, it can be assessed the extent of peroxidation damage in tissues. It can also indirectly indicate the extent of tissue peroxidation damage. Total antioxidant capacity (T-AOC) comprises numerous antioxidant substances and enzymes. It can be utilized to evaluate antioxidant capacity in kidney tissues. Cd induces OS by increasing the level of the oxidative product MDA, which inhibits the antioxidant enzyme activity and depletes the GSH activity (Ge et al., 2019). Zhuang et al. (2019) found that Cd led to elevated MDA levels in duck kidney tubular epithelial cells and decreased the activities of CAT, SOD, T-AOC, and GSH, which resulted in OS.

Endoplasmic reticulum (ER) stress plays an important role in organismal toxicity induced by Cd (Chen et al., 2019; Wang et al., 2023). Under stimulation, the ER struggles to bear the load of folded or misfolded proteins, which leads ER stress. Glucose-regulating protein 78 (GRP78) is an important regulator of ER stress. During ER homeostatic equilibrium, GRP78 binds to ER stress receptors, including protein kinase RNA-like ER kinase (PERK), inositol-requiring protein 1 (IRE1), and activating transcription factor 6 (ATF6), which causes the inhibition of the unfolded protein response (UPR). By contrast, during ER stress, GRP78 bind to misfolded proteins and separates from ER stress receptors (Kawaguchi et al., 2020). This action activates eukaryotic translation initiation factor 2 (eIF2α), transcription factor 4 (ATF4), and signaling molecules downstream of the C/EBP homologous protein (CHOP). Multiple researchers have found that Cd induces ER stress after exposure to the testes, heart, and ovaries (Wan et al., 2018; Wang et al., 2023; Zhu et al., 2024).

Autophagy is a complex process of degrading harmful components within cells through lysosomes and is considered a form of programmed cell death. Autophagy can be divided into macroautophagy, chaperone-mediated autophagy, and microautophagy. The most common is macroautophagy, so it is often considered a representative of autophagy. The core process of autophagy is the formation of autophagosomes, which is regulated by autophagy-related genes (Atg) and has great significance (Nishimura and Tooze, 2020). Studies have shown that Cd can inflict organ damage through autophagy (Ma et al., 2022).

Flavonoids are natural organic compounds that exist in nature and have antioxidant, antibacterial, anti-inflammatory, and myocardial protective effects (Chen et al., 2023). Naringenin (Nar) is a dihydroflavonoid extensively found in citrus plants. It exerts pharmacological effects such as antioxidation, anti-inflammation, anti-injury, and kidney protection and is widely used in food and medicine (Wang et al., 2012; Wang et al., 2017). The antioxidant activity of Nar is primarily demonstrated in scavenging free radicals, preventing lipid peroxidation, and enhancing the body’s antioxidant activity by reducing reactive oxygen species (ROS) production (Lu et al., 2022). Most studies have shown that Nar has a kidney-protective effect. Nar can alleviate the kidney damage caused by CCl_4_ in rats and ease the kidney damage in rats with gestational diabetes mellitus (Zhao et al., 2020; Ammar et al., 2022). However, the research on the amelioration of cadmium chloride (CdCl_2_)-induced kidney injury by Nar in chickens is limited.

CdCl_2_ tends to accumulate in living organisms, so it poses a hazard to the poultry industry and to humans who consume Cd-contaminated poultry. Thus, mitigating the associated risks is essential. Nar, as a natural organic compound, can alleviate the damage caused by Cd through its biological activity. In the present study, CdCl_2_ and Nar were supplemented into diets to investigate the effects of Nar on CdCl_2_-induced renal injury in chickens and its mechanisms, including OS, ER stress, and autophagy.

## 2 Materials and methods

### 2.1 Chemicals and reagents

Cadmium chloride (CdCl_2_, 99.95% purity) (CAS No. 7790-78-5, Molecular weight: 228.36) and Naringenin (Nar, 97% purity) (CAS No. 67604-48-2, Molecular weight: 272.26) were procured from Shanghai Aladdin Biochemical Technology Co., Ltd. (Shanghai, China). Uric acid (UA, C012-2-1), Blood Urea Nitrogen (BUN, A013-2-1), Creatinine (CAT, A007-1-1), β-N-Acetylglucosaminidase (NAG, A031-1-1), Superoxide Dismutase (SOD, A001-2-2), Catalase (CAT, A007-1-1), Glutathione (GSH, A006-2-1), Total antioxidant capacity (T-AOC, A015-3-1) and Malondialdehyde (MDA, A003-1-2) kits were selected from Nanjing Jiancheng Institute of Biological Engineering (Nanjing, China).

### 2.2 Animal experiment

Sixty 1-day-old male Hy-Line White chickens that passed quality inspection were evenly divided into the control group (standard diet), 150 mg/kg CdCl_2_ group (standard diet supplemented with CdCl_2_), 250 mg/kg Nar group (standard diet supplemented with Nar), and 150 mg/kg CdCl_2_ + 250 mg/kg Nar co-treatment group (standard diet supplemented with CdCl_2_ and Nar), to establish an animal model of chronic Cd poisoning. Each group of 15 animals was kept in cages with ample space for movement, suitable temperature, full sunlight, and fresh air (Size: 160 cm × 80 cm × 40 cm). They had free access to feed and water. The standard diet used in the experiment was purchased from Mengjin County Fanda Feed Factory in China (Table 1 shows the nutritional content). The applied doses of CdCl_2_ and Nar were based on previous preexperiments. After 8 weeks of treatment, the chickens were euthanized (exsanguination following ether anesthesia), collect neck blood. The abdominal cavity was opened with sterile scissors to remove the kidneys, which is located on both sides of the sacrum and in the kidney fossa of the ilium.

**TABLE 1:** Nutrient content of chickens standard diet.

Nutrients	Concentrations (%)
Crude protein (≥)	16.0
Crude fibre (≤)	8.0
Crude ash (≤)	15.0
Calcium	3.3–4.5
Total phosphorus (≥)	0.4
Sodium chloride	0.3–0.8
Lysine (≥)	0.85
Methionine	0.3–0.9
Moisture (≤)	14.0

### 2.3 Body weight and relative kidney weight

Chickens were weighed weekly, and their weight data were recorded. At the end of the test, the chickens were euthanized, the kidneys were removed, weighed, and recorded.

Relative kidney weight = Kidney weight/Chicken weight × 100%

### 2.4 Determination of kidney function and antioxidant indexes

Blood was collected from the necks of chickens and stored in test tubes. Then, the blood samples were centrifuged at 3,000 rpm and 4°C, and the serum was collected. Kidney function indexes, such as Uric acid (UA), Blood urea nitrogen (BUN), Creatinine (Cr), and β-N-acetyl-glucosaminidase assay kit (NAG), were measured. A total of 0.1 g kidney tissue was collected and added with nine times normal saline. Afterward, the kidney tissue was ground at a low temperature for 90 s and centrifuged at 3,000 rpm and 4°C for 10 min. The supernatant was collected to detect antioxidant indexes, including CAT, T-AOC, MDA, SOD, and GSH. All the necessary kits were purchased from Nanjing Jiancheng Bioengineering Institute (Nanjing, China). Among them, UA, Cr, T-AOC, MDA, and GSH were determined by the enzyme-labeling method by through a Tecan instrument (model: Infinite M Nano, Swiss Confederation). BUN, NAG, CAT, and SOD levels were determined using a spectrophotometer with MAPADA (model: UA-1800PC, Shanghai).

### 2.5 Pathological section analysis of kidney

Pathological sections of the kidney were stained with hematoxylin-eosin staining (H&E). A small piece of kidney tissue was cut into 4 μm tissue blocks and fixed in 4% paraformaldehyde for 24 h. After rinsing with running water three times for 5 min each time, the samples were dehydrated with ethanol to varying concentrations for 45 min (30%, 50%, 70%, 95%, and 100%). The tissue blocks were initially immersed in a mixture of ethanol and xylene, followed by pure xylene to render the kidney tissue transparent. After paraffin embedding, and the tissue sections embedded were transferred onto slides at 60°C and dried overnight using a microtome. The treated sections were then dewaxed and rehydrated, colored with H&E staining solution, and observed a microscopy.

### 2.6 Detection of renal-cell apoptosis by TUNEL method

The prepared kidney paraffin sections were soaked in xylene twice for 5 min each time. They were then treated with gradient ethanol once for 3 min each time. The tissues were then treated with Proteinase K for 30 min and washed with PBS. After preparing the TUNEL reaction mixture, 50 μL was added to the specimen, the mouth film was sealed in a dark, wet box, and the reaction was carried out at 37°C for 60 min. After adding to the termination reaction buffer and rinsing three times with PBS, DAB substrate was added to the specimen, reacted at 25°C for 10 min, and rinsed. Hematoxylin was redyed and washed with distilled water. After gradient dehydration in alcohol and xylene, the sample was sealed for transparency. Apoptotic cells were observed using an optical microscope and photographed.

### 2.7 Quantitative real-time PCR (RT-qPCR) detection

The kidney tissue, frozen at −80°C, was cut into pieces the size of mung beans. They were then placed into a 1.5 mL enzyme-free tube containing 800 µL of TRIgene (Genstar, Beijing) and ground using a homogenizer for 60 s. After adding 200 μL of chloroform, mixing well, and centrifuging for 15 min, the supernatant was collected. An equal amount of isopropyl alcohol was added, centrifugation was performed again for 10 min, and the supernatant was discarded. We added 75% anhydrous ethanol to the samples, washed them twice, and centrifuged them for 15 min each time. Anhydrous ethanol was removed by suction, the RNA was dried, and 30 μL of enzyme-free water was added to mix thoroughly for the determination of nucleic acid concentration. The proposed RNA was reverse transcribed into cDNA by using StarScript III All-in-one RT Mix with gDNA Remover kit (Genstar, Beijing). Finally, according to the reaction system (20 μL) and conditions specified in the RT-qPCR instructions, three techniques were repeated for each gene. Using β-actin as the internal reference, BIO-RAD fluorescence quantitative PCR (model: C1000 Touch™ Thermal Cycle, United States) was used to obtain the results. The expression of target genes was calculated by the 2^−ΔΔCt^ method (Table 2).

**TABLE 2:** Fluorescent quantitative PCR primer sequence.

Gene	Target gene sequences (5’ → 3′)	TM (°C)	Product length	Gen bank No.
*GRP78*	F: ATCAGCCCACTGTGACCATT R: AGCAGGAGGGATTCCAGTCA	F = 56.6 R = 58.3	101bp	XM_046928788.1
*PERK*	F: TCATCCAGCCTCAGTAAACC R: ACAACATCCTCGCCCAGT	F = 54.2 R = 56.8	164bp	XM_040671515.2
*eIF2α*	F: CCCAGTTTCACAGAGAGCG R: CCTGCTGTGCCATCTTTG	F = 56.2 R = 54.4	248bp	XM_046923980.1
*ATF4*	F: GTTTTTCAAGGCACCGCACA R: ACTTTCCTCAGCCACCGAAC	F = 56.9 R = 57.9	176bp	XM_046906693.1
*CHOP*	F: GGCCTGGTTCAATATGGGGA R: AATGTCTGCATAGGACACTGGT	F = 57.5 R = 55.8	182bp	XM_046934870.1
*ATF6*	F: TAGCAAGGAGCCCAGCC R: GCCTTTGTGACTCTGTTCG	F = 59.0 R = 59.8	185bp	XM_040677276.2
*LC3*	F: GACTTGTAGAGGCTTTCCTTCATAG R: GACTTCATCTTCAGCAGAACACTAC	F = 54.7 R = 55.2	191bp	NM_001006279.3
*P62*	F: GCGGCGAGAAGGAGATG R: GCAGCAGGGGCAGTCAA	F = 56.6 R = 59.4	194bp	XM_040682727.2
*Beclin-1*	F: AAGTGTGGAGAACCAGATGCG R: GCAAGGGCATGCAGTAACAG	F = 57.7 R = 57.3	210bp	NM_001006332.1
*ATG5*	F: AGAGATGTGTGGTTTGGACGC R: GCCGAGGAAGGGCTGTATT	F = 57.9 R = 57.8	271bp	XM_046914044.1
*β-actin*	F: CCGCTCTATGAAGGCTACGC R: CTCTCGGCTGTGGTGGTGAA	F = 58.6 R = 60.3	128bp	NM_205518.2

### 2.8 Western blot

The lysate was prepared using phosphatase inhibitor mixture A (Cat: No. P1081, Beyotime), protease inhibitor mixture (Cat: No. P1010, Beyotime) and RIPA lysate (strong) (Cat: G2002, Servicebio). A total of 400 μL prepared lysate was added to 40 mg kidney tissue for complete cleavage and protein extraction. A BCA kit was used in the assessment of protein content. The protein was denatured, added to the prepared 12% gel and then transferred to a 0.45 μm PVDF membrane (4 cm × 8.5 cm) using SDS-PAGE electrophoresis. Skimmed milk powder was incubated, then the appropriate concentration of primary antibodies was selected: LC3 (1:1,000, 2775S, Cell Signaling), GRP78 (1:6,000, 11587-1-AP, Proteintech), PERK (1:500, 24390-1-AP, Proteintech), CHOP (1:1,500, 15204-1-AP, Proteintech), ATF4 (1:800, 10835-1-AP, Proteintech), ATF6 (1:5,000, 24169-1-AP, Proteintech), eIF2α (1:1,000, ab169528, Abcam), P62 (1:1,000, PM066, MBL), Beclin-1 (1:4,000, 11306-1-AP, Proteintech), and ATG5 (1:1,000, 10181-2-AP, Proteintech) for 12–18 h. The secondary antibody was incubated for 50 min, and washed with TBST three times in between. Aplegen chemiluminescence instrument (Model: Omega Lum G, United States) was utilized for exposure and analysis of gray values.

### 2.9 Statistical analysis

SPSS 26.0 was used to analyze the differences in experimental results (one-way ANOVA and LSD), and GraphPad Prism 8 was used for to plot data. A *P*-value less than 0.05 was considered statistically significant (*P* < 0.05). Values are expressed as the mean ± SEM.

## 3 Results

### 3.1 Effect of Nar and CdCl_2_ on chicken weight

Nar showed no significant effect on the weights of chickens (Table 3). Compared with the control group, the body weights of chickens exposed to CdCl_2_ exhibited significant differences starting from the fourth week, with decreases of 41.49%, 40.24%, 41.07%, 40.86%, and 40.58% (*P*< 0.01). However, compared with the CdCl_2_ group, the body weights of CdCl_2_ + Nar co-treatment group increased by 13.51%, 12.51%, 13.80%, 16.15%, and 18.28% (*P*< 0.05) from week 4. Nar reduced CdCl_2_-induced weight loss in chickens, and this effect gradually increased over time.

**TABLE 3:** Effect of Nar and CdCl_2_ on the body weight of chickens.

Days of treatment	Control	CdCl_2_	Nar	CdCl_2_ + Nar
0 week	49.78 ± 4.41	50.4 ± 1.46	48.18 ± 3.2	49.13 ± 1.44
1 week	107.98 ± 8.36	77.02 ± 5.97	106.15 ± 10.63	91.8 ± 12.69
2 weeks	191.77 ± 21.77	147.02 ± 15.94	200.18 ± 22.59	169.82 ± 17.95
3 weeks	289.43 ± 9.68	189.33 ± 21.69	309.02 ± 23.78	211.17 ± 37.54
4 weeks	459.3 ± 13.19	268.72 ± 47.7^**^	461 ± 12.26	305.03 ± 16.58^#^
5 weeks	632.83 ± 19.85	378.17 ± 52.66^**^	634.83 ± 26.08	425.5 ± 33.54^#^
6 weeks	840 ± 17.89	495 ± 63.8^**^	828.33 ± 29.27	563.33 ± 55.02^#^
7 weeks	1011.67 ± 43.09	598.33 ± 96.21^**^	1008.33 ± 37.64	695 ± 75.03^#^
8 weeks	1211.67 ± 56.01	720 ± 101.39^**^	1176.67 ± 57.15	851.67 ± 51.15^##^

Values are expressed as the mean ± SEM. n = 6.

*P* < 0.01: compared with the control group.

*P* < 0.05,

*P* < 0.01: compared with the CdCl_2_ group.

### 3.2 Effect of CdCl_2_ and Nar on kidney weight of chickens

Detection of the relative weight of chicken kidney organs (Table 4). The relative kidney weight of CdCl_2_ was significantly higher than that of the control group, showed an increase of 96.67% (*P* < 0.01). Compared with the CdCl_2_ group, the CdCl_2_ + Nar co-treatment group presented significantly reduced relative kidney weight of chickens by 16.95% (*P* < 0.05).

**TABLE 4:** Relative kidney weight results after 8 weeks of treatment.

Treatment	Relative kidney weight
Control group	0.6 ± 0.06
CdCl_2_ group	1.18 ± 0.19^**^
Nar group	0.59 ± 0.07
CdCl_2_ + Nar group	0.98 ± 0.15^#^

Values are expressed as the mean ± SEM. n = 6.

*P* < 0.01: compared with the control group.

*P* < 0.05: compared with the CdCl_2_ group.

### 3.3 Effects of CdCl_2_ and Nar on kidney function in chickens

**FIGURE 1:**
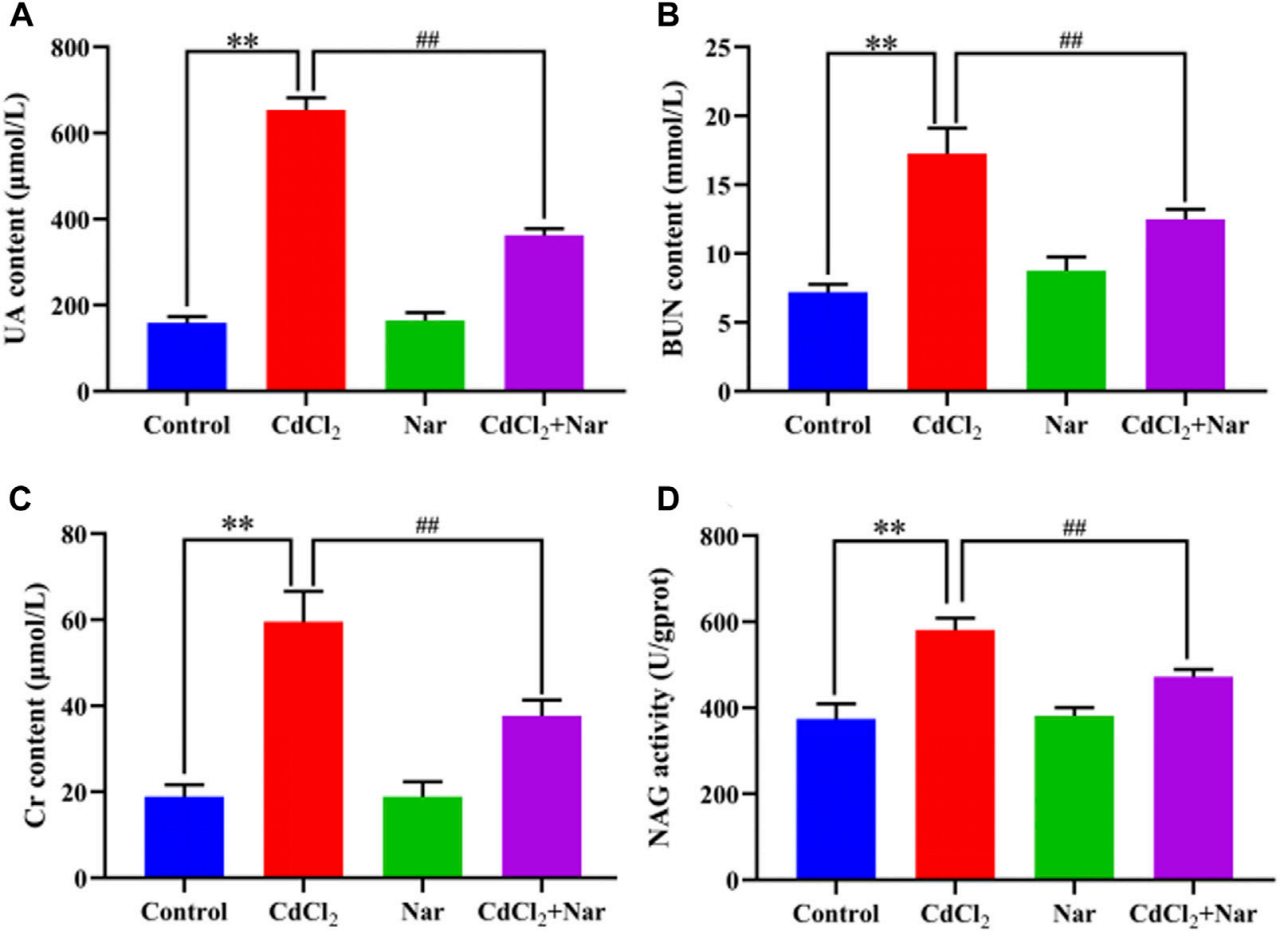
Effects of CdCl_2_ and Nar on kidney function in chickens. **(A)** UA, **(B)** BUN, **(C)** Cr, **(D)** NAG. n = 6. Values are expressed as the mean ± SEM. ^**^ *P* < 0.01: compared with the control group. ^##^ *P* < 0.01: compared with the CdCl_2_ group.

The kidney function indicators UA, BUN, CRE, and NAG were tested in the investigation of the effect of CdCl_2_ on kidney damage in chickens (Figure 1). Compared with the control group, the levels and activities of serum UA (312.11%), BUN (140.04%), Cr (215.02%), and NAG (55.27%) in the Cd group were significantly increased (*P* < 0.01). The levels and activities of UA (44.55%), BUN (27.64%), Cr (36.66%), and NAG (18.71%) in the serum of chickens from the CdCl_2_ + Nar co-treatment group were significantly lower (*P* < 0.05) than those in the CdCl_2_ group. This study indicates that Nar alleviated CdCl_2_-induced kidney impairment in chickens.

### 3.4 Effects of CdCl_2_ and Nar on kidney oxidation indexes in chickens

**FIGURE 2:**
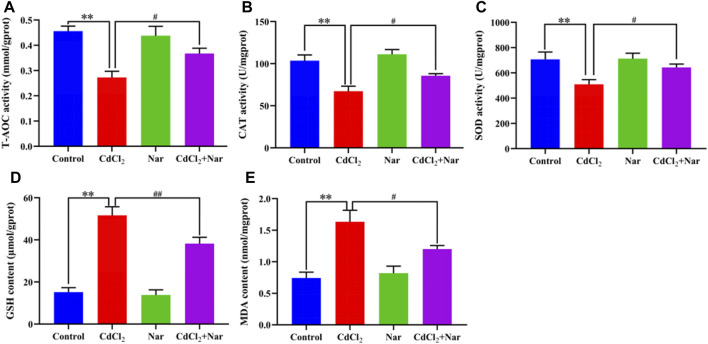
Effects of CdCl_2_ and Nar on kidney antioxidant levels in chickens. **(A)** T-AOC, **(B)** CAT, **(C)** SOD, **(D)** GSH, **(E)** MDA. n = 6. Values are expressed as the mean ± SEM. ^**^ *P* < 0.01: compared with the control group. ^#^ *P* < 0.05, ^##^ *P* < 0.01: compared with the CdCl_2_ group.

Antioxidant indexes, including CAT, T-AOC, MDA, SOD, and GSH, were detected in the assessment of Cd-induced OS in the kidneys (Figure 2). Compared with the control group, the levels of T-AOC (20.51%), CAT (35.05%), and SOD (27.94%) of the CdCl_2_ group were significantly decreased, whereas the levels of GSH (241.01%) and MDA (120.05%) were significantly increased (*P* < 0.01). In addition, compared with the CdCl_2_ group, significantly higher activities of T-AOC (9.58%), CAT (27.36%), and SOD (26.20%) were observed in the CdCl_2_ + Nar co-treatment group, and the levels of GSH (26.04%) and MDA (26.58%) were significantly decreased (*P* < 0.05). Nar alleviated CdCl_2_-induced OS in chicken kidneys through its antioxidant effect.

### 3.5 Observation of a kidney pathological section

**FIGURE 3:**
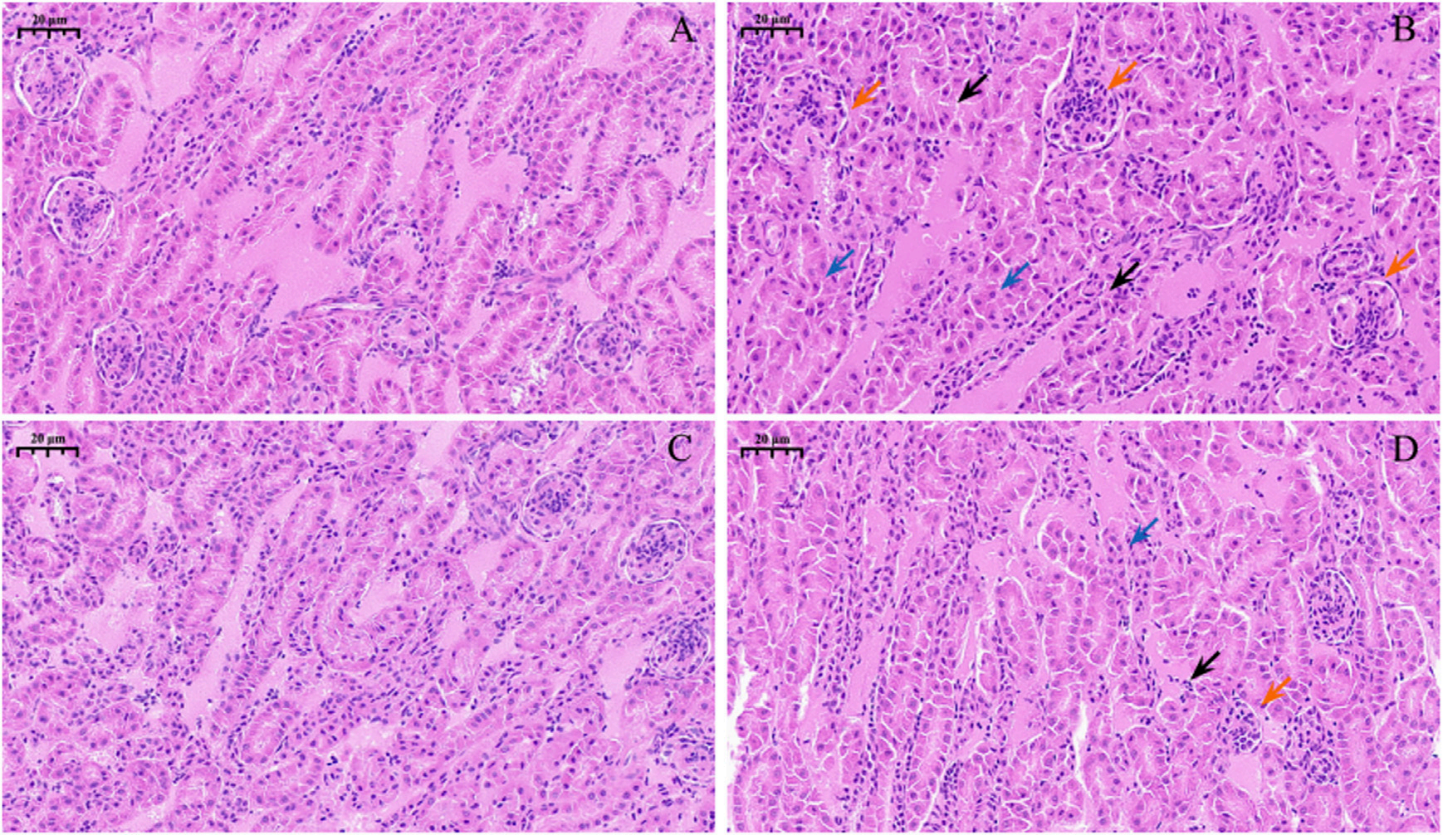
Histopathological observation of Nar intervention on the kidneys of chickens with chronic CdCl_2_ poisoning. **(A)** control group, **(B)** CdCl_2_ group, **(C)** Nar group, **(D)** CdCl_2_ + Nar group. The scale is 20 μm. Red arrow: blurred and irregular glomerular boundaries; blue arrow: nucleus is dissolved; black arrow: boundary before the renal tubules is unclear.

Observations of pathological section showed that the control group (Figure 3A) and Nar group (Figure 3C) had clear renal-tubule boundaries and normal glomerular structure. Renal injury was severe in the CdCl_2_ group (Figure 3B), with blurred and irregular glomerular boundaries (red arrow), as well as dissolved nucleus (blue arrow). The morphology of the renal tubules was incomplete, and the boundary before the renal tubules was unclear (black arrow). In the CdCl_2_ + Nar group (Figure 3D), renal tissue injury significantly improved.

### 3.6 Renal cell apoptosis was detected using the TUNEL method

**FIGURE 4:**
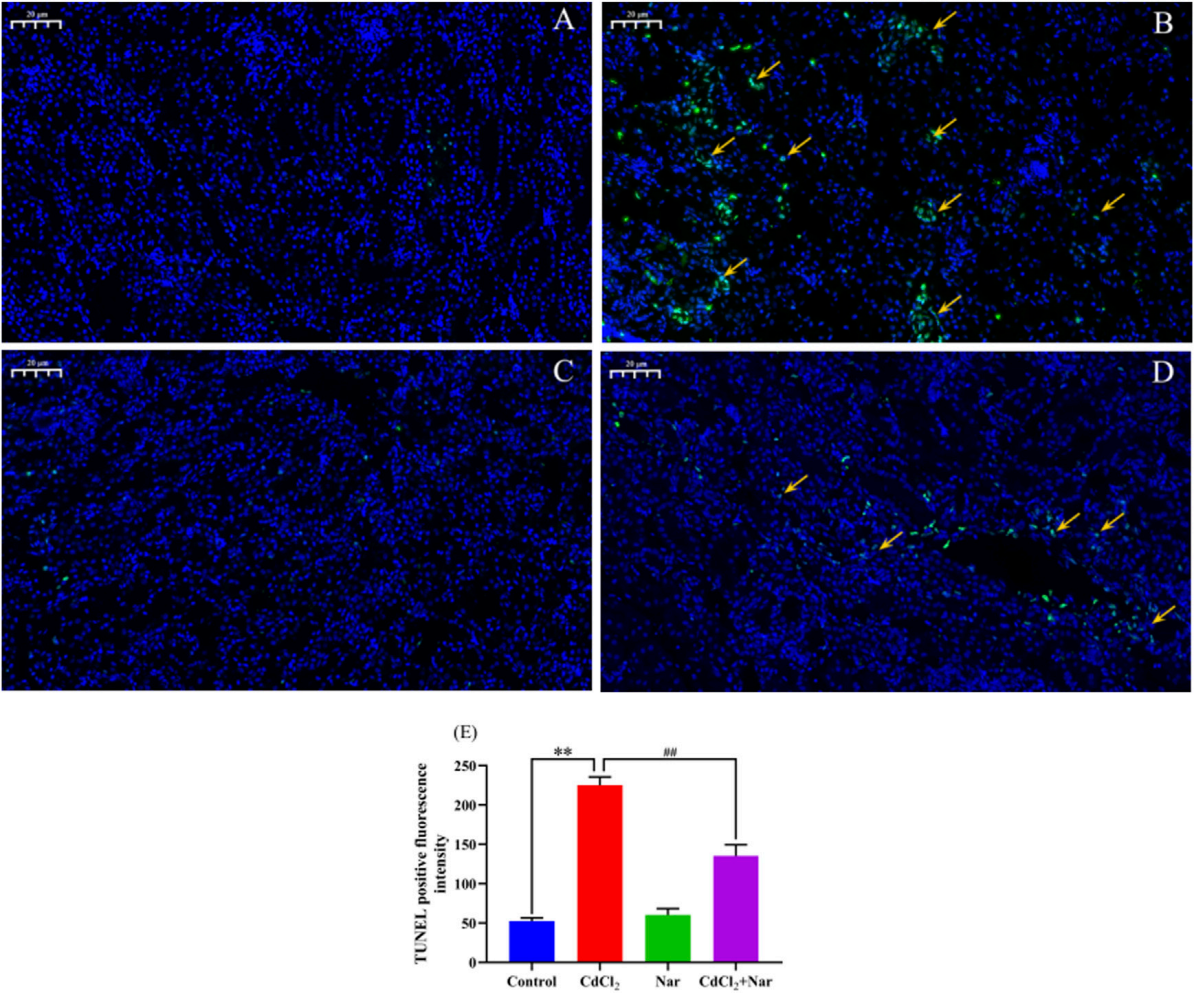
Effects of CdCl_2_ and Nar on the apoptosis of chicken renal cells. **(A)** control group, **(B)** CdCl_2_ group, **(C)** Nar group, **(D)** CdCl_2_ + Nar group. The scale is 20 μm. Orange arrow: TUNEL apoptotic cells. **(E)** TUNEL positive fluorescence intensity. ^**^ *P* < 0.01: compared with the control group. ^#^ *P* < 0.05, ^##^ *P* < 0.01: compared with the CdCl_2_ group.

To verify the effects of CdCl_2_ and Nar on the apoptosis of chicken renal cells, TUNEL staining was used to detect apoptosis in each group (Figure 4). The green fluorescence intensity of the control group (Figure 4A) and Nar group (Figure 4C) was similar, but the difference was not significant (*P* > 0.05). However, in the CdCl_2_ group (Figure 4B), the green fluorescence intensity was significantly enhanced compared with those of control group and Nar group (*P* < 0.01). In the CdCl_2_ + Nar group (Figure 4D), the green fluorescence intensity was significantly weaker than that in the CdCl_2_ group (*P* < 0.01). Analysis of TUNEL-positive fluorescence intensity in each group (Figure 4E).

### 3.7 Effects of CdCl_2_ and Nar on mRNA expression levels of ER stress-related genes in chicken kidneys

**FIGURE 5:**
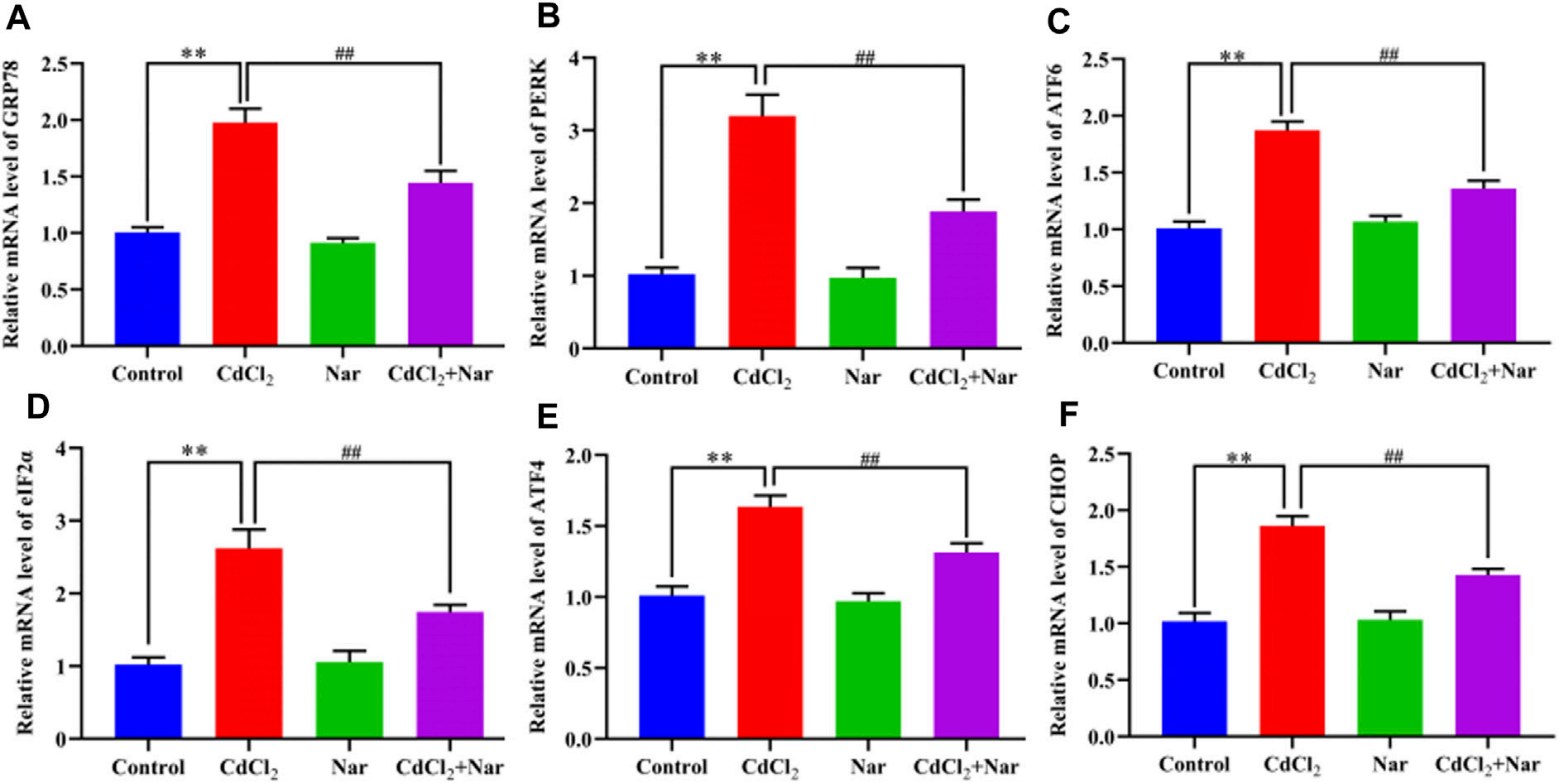
Effects of CdCl_2_ and Nar on the expression levels of ER stress-related genes. **(A)** GRP78, **(B)** PERK, **(C)** ATF6, **(D)** eIF2α, **(E)** ATF4, **(F)** CHOP. Values are expressed as the mean ± SEM. ^**^ *P* < 0.01: compared with the control group. ^##^ *P* < 0.01: compared with the CdCl_2_ group.

We tested the mRNA expression levels of ER stress-related genes, to verify the effects of CdCl_2_ and Nar on ER stress (Figure 5). Results showed that the mRNA expression levels of GRP78 (96.68%), PERK (213.24%), ATF6 (85.61%), eIF2α (156.34%), ATF4 (61.74%), and CHOP (82.9%) in the CdCl_2_ group was significantly higher than that in the control group (*P* < 0.01). The mRNA expression levels of the GRP78 (26.9%), PERK (41.08%), ATF6 (27.41%), eIF2α (33.49%), ATF4 (19.74%), and CHOP (23.22%) in the CdCl_2_ + Nar group was significantly lower than that in the CdCl_2_ group (*P* < 0.01). No significant difference existed between the control group and Nar group (*P* > 0.05).

### 3.8 Effects of CdCl_2_ and Nar on ER stress-related proteins in chicken kidneys

**FIGURE 6:**
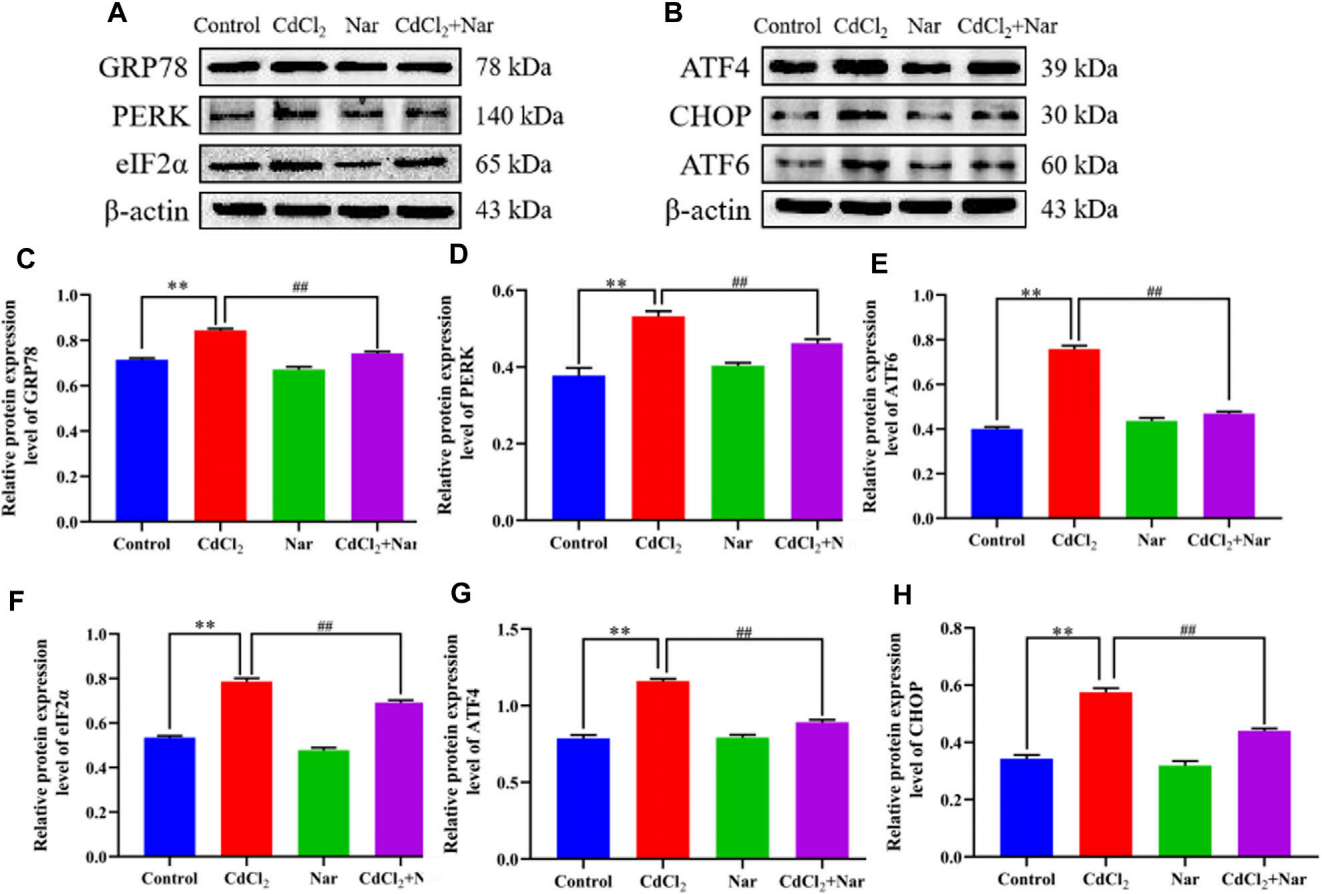
Effects of CdCl_2_ and Nar on ER stress-related proteins. **(A)** Western blot analysis of GRP78, PERK, eIF2α, and β-actin proteins. **(B)** Western blot analysis of ATF4, CHOP, and ATF6 proteins. **(C)** GRP78, **(D)** PERK, **(E)** ATF6, **(F)** eIF2α, **(G)** ATF4, **(H)** CHOP. Values are expressed as the mean ± SEM. ^**^ *P* < 0.01: compared with the control group. ^#^ *P* < 0.05, ^##^ *P*< 0.01: compared with the CdCl_2_ group.

To further verify the effects of CdCl_2_ and Nar on ER stress, we examined the expression levels of ER stress-related proteins (Figure 6). Results showed that the proteins expression levels of GRP78 (18.02%), PERK (55.05%), ATF6 (72.18%), eIF2α (36.09%), ATF4 (41.12%), and CHOP (51.86%) in the CdCl_2_ group were significantly higher than those in the control (*P* < 0.01). The proteins expression levels of GRP78 (11.91%), PERK (25.14%), ATF6 (22.23%), eIF2α (7.23%), ATF4 (16.34%), and CHOP (22.31%) in the CdCl_2_ + Nar co-treatment group was significantly lower than that in the CdCl_2_ group (*P* < 0.01). The results indicate that CdCl_2_ activates ER stress in chicken kidneys, whereas Nar alleviates CdCl_2_-induced ER stress.

### 3.9 Effects of CdCl_2_ and Nar on mRNA expression levels of autophagy-related genes in chicken kidneys

**FIGURE 7:**
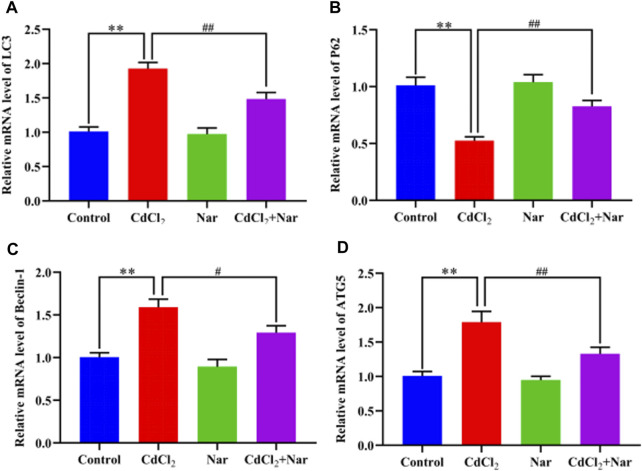
Effects of CdCl_2_ and Nar on the expression levels of autophagy-related genes. **(A)** LC3, **(B)** P62, **(C)** Beclin-1, **(D)** ATG5. Values are expressed as the mean ± SEM. ^**^ *P* < 0.01: compared with the control group. ^#^ *P* < 0.05, ^##^ *P* < 0.01: compared with the CdCl_2_ group.

The mRNA expression levels of autophagy genes such as Microtubule-associated protein light chain 3 (LC3), Sequestosome 1 (P62), Beclin-1, and Autophagy-related gene 5 (ATG5) were detected to verify the effects of CdCl_2_ and Nar on autophagy (Figure 7). Results showed that the mRNA expression levels of the autophagy genes LC3 (90.49%), Beclin-1 (58.12%), and ATG5 (77.21%) in the CdCl_2_ group were significantly higher than those in the control group (*P* < 0.01). Additionally, the mRNA expression level of P62 (48.02%) were significantly decreased (*P* < 0.01). Conversely, compared with the CdCl_2_ group, the mRNA expression levels of autophagy genes LC3 (23.05%), Beclin-1 (18.74%), and ATG5 (25.73%) in the CdCl_2_ + Nar group decreased, whereas the expression level of P62 (57.31%) was significantly increased (*P* < 0.05). No significant difference existed between the control group and Nar group (*P* > 0.05).

### 3.10 Effect of CdCl_2_ and Nar on autophagy-related proteins in chicken kidneys

**FIGURE 8:**
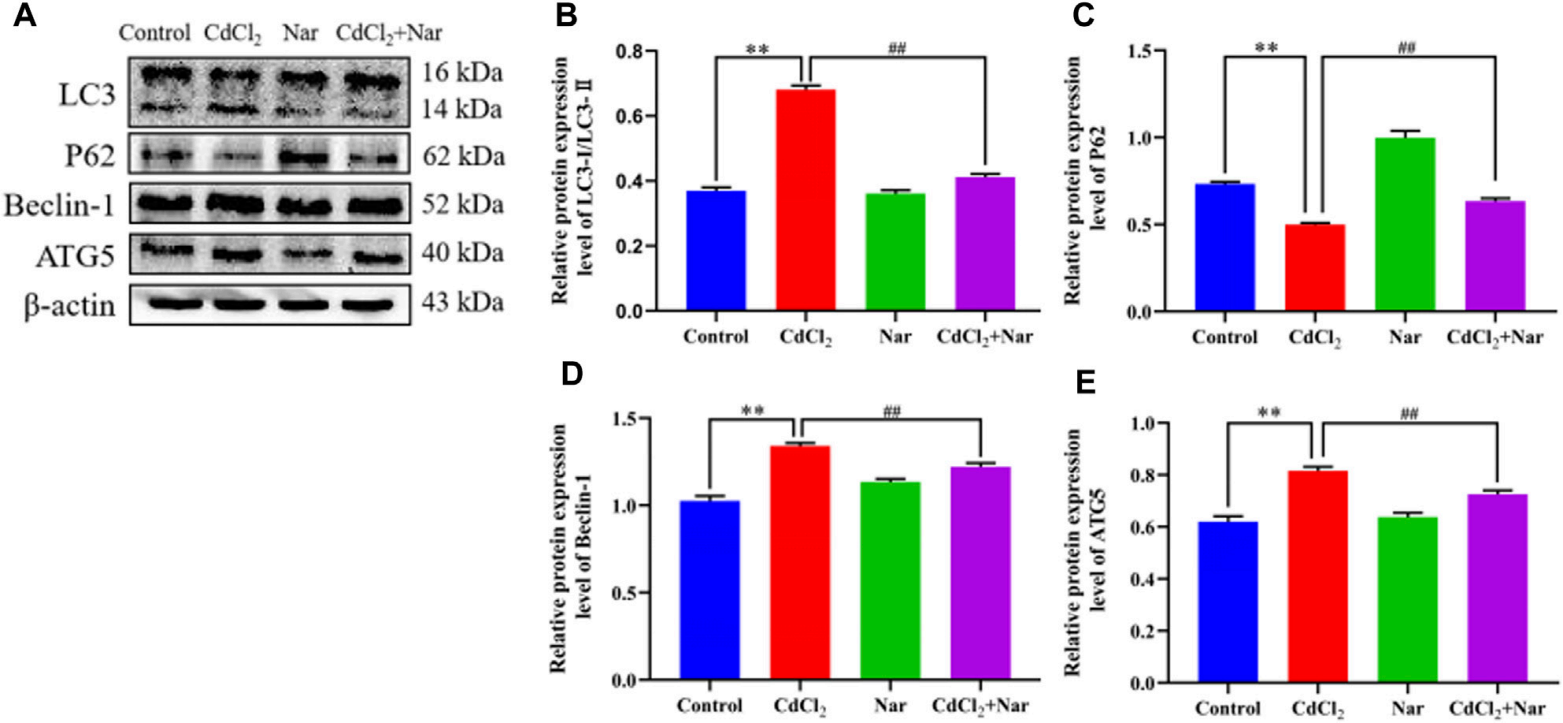
Effects of CdCl_2_ and Nar on autophagy-related proteins. **(A)** Western blot analysis of LC3, P62, Beclin-1, ATG5, and β-actin proteins. **(B)** LC3-Ⅱ/LC3-Ⅰ, **(C)** P62, **(D)** Beclin-1, **(E)** ATG5. Values are expressed as the mean ± SEM. ^**^ *P* < 0.01: compared with the control group. ^##^ *P* < 0.01: compared with the CdCl_2_ group.

The expression levels of autophagy-related proteins were detected to further verify the effects of CdCl_2_ and Nar on autophagy (Figure 8). The results revealed that the significantly higher levels of autophagy-related proteins LC3 (78.75%), Beclin-1 (17.88%), and ATG5 (27.15%) and the significantly lower expression level of P62 (28.07%) in the CdCl2 group than those in the control group (*P* < 0.01). The CdCl2 + Nar co-treatment group showed significantly lower expression levels of autophagy-related proteins LC3 (38.08%), Beclin-1 (10.05%), and ATG5 (11.62%) and significantly increased expression level of P62 (27.93%) compared with those in the CdCl2 group (*P* < 0.01). These results indicate that CdCl2 activates renal autophagy in chickens, and Nar can alleviate CdCl_2_-induced autophagy.

## 4 Discussion

Cd is an environmental pollutant that enters the bodies of humans and animals through the air, drinking water, and food chain. It travels through the bloodstream to organs throughout the body and accumulates in the kidneys. Toxicological studies reveal that the relative organ weight plays an important role in evaluating toxicant effects *in vivo*. The ratio of organ weight to body weight can be calculated to reflect the toxic effects of Cd on organs (Gebrezgi et al., 2020). Studies have shown that Cd treatment can cause kidney dysfunction (Gong et al., 2022; Lian et al., 2023). BUN, UA, and Cr are commonly used indicators of kidney function. NAG is a glycosidase found in renal proximal tubules and mainly used in the detection of acute and chronic kidney diseases (Novak et al., 2023). Most researchers have found that CdCl_2_ poisoning induces substantial increases in renal UA, BUN, and Cr levels (Kim et al., 2018; Salama et al., 2021; Huang et al., 2022). Śliwińska-Mossoń et al. found that long-term Cd exposure in rats resulted in increased NAG activity in the kidneys (Śliwińska-Mossoń et al., 2019). Our findings are consistent with those of previous research, which showed that Cd treatment caused a decrease in the body weight of chickens and an increase in relative weight of their kidneys. CdCl_2_ treatment considerably increased the levels and activities of UA, BUN, Cr, and NAG. The Cd + Nar co-treatment group exhibited the remarkable alleviation of the weight loss and kidney injury induced by CdCl_2_. These results imply that Nar imply alleviate the renal toxicity induced by CdCl_2_.

OS is one of the many mechanisms underlying kidney injury caused by Cd. Studies have shown that exposure to Cd leads to the excessive production of ROS by the mitochondria and NADPH oxidase, ultimately causing OS (Yan and Allen, 2021). Sun et al. found that Cd treatment substantially elevated the MDA content in duck liver, while reducing SOD activity (Ge et al., 2019). Zhu et al. found that the levels of MDA and GSH in the kidneys of laying hens increase upon adding CdCl_2_ to the diet (Zhu et al., 2019). Consistent with previous studies, the present research revealed that exposure to CdCl_2_ induced OS in chicken kidneys, as proven by decreased T-AOC, SOD, and CAT levels and elevated MDA and GSH levels. Nar has a powerful antioxidant effect, and it can alleviate OS in the livers of adriamycin-poisoned rats, which led to increased SOD and CAT activities (Wali et al., 2020). Das et al. found that Nar alleviated the liver and renal toxicity of Cd in albino mice by increasing the levels of antioxidant indices, such as SOD and CAT (Das et al., 2016). In this experiment, the CdCl_2_ + Nar co-treatment group exhibited significantly higher activities of T-AOC, SOD, and CAT and significantly lower MDA and GSH levels compared with the CdCl_2_ group. Therefore, Nar can reduce CdCl_2_-induced OS in the kidney.

In histology and pathology, H&E staining plays a key role in clearly showing the structural differences between normal and abnormal tissues, as well as in identifying pathological changes in tissues (Feldman and Wolfe, 2014). After exposure to Cd in pregnant rats, the fetal kidneys were damaged, and the renal histopathological manifestations included renal tubular injury (Jacobo-Estrada et al., 2016). Apoptosis also plays an important role in Cd-induced nephrotoxicity (Li et al., 2022). In the current work, H&E and TUNEL methods were used to investigate the impact of CdCl_2_ and Nar on renal tissue injury and apoptosis in chickens. Results showed that the fluorescence intensity of the Nar group was similar to that of the control group, and the glomerular structure appeared normal based on H&E staining. The fluorescence intensity of the CdCl_2_ group was significantly higher than that of the control group and Nar group. Meanwhile, kidney injury was significantly aggravated, characterized by blurred and irregular glomerular boundaries. This finding indicated that CdCl_2_ may induce kidney injury and apoptosis in chickens, consistent with the findings of Li et al. (2022). Furthermore, apoptosis significantly decreased in the CdCl_2_ + Nar group, and kidney injury notably improved. These results suggested that Nar alleviated CdCl_2_-induced renal injury and apoptosis in chickens.

OS disrupts the ER organelles, which results in the overproduction of intracellular ROS. Such a phenomenon causes erroneous protein aggregation, which in turn induces ER stress and triggers UPR (Zhang et al., 2023). Studies have shown that the heavy metal Cd can cause ER stress *in vivo* and *in vitro* (Kitamura and Hiramatsu, 2010). He et al. (2022) observed that Cd activated liver ER stress in a mouse model of Cd treatment. Wang et al. found that Cd treatment upregulates GRP78, ATF6, IRE1, CHOP, PERK, and JNK proteins in chicken testis (M. Wang et al., 2023). Consistent with the abovementioned results, we found that ER stress-related proteins, such as GRP78, ATF6, CHOP, PERK, ATF4, and eIF2α, can be upregulated by the addition of CdCl_2_ diet to chickens. Meanwhile, the CdCl_2_ + Nar co-treatment group showed reduced expression levels of ER stress-related proteins. Nar significantly reduced the CCl_4_-induced elevations of CHOP, GRP78, ATF6, ATF4, IRE1, PERK, and XBP1s in rat liver (Ustuner et al., 2020). This result indicates that Nar alleviates CdCl_2_-induced renal ER stress in chickens.

The autophagy process is crucial to maintain balance in the intracellular environment, with LC3, P62, Beclin-1, and ATG5 serving as key factors. During autophagy, LC3 is a marker of autophagy and is categorized into LC3-I and LC3-II (Kabeya et al., 2000). The LC3-II-to-LC3-I ratio is commonly used to indicate the LC3 level. P62 is a multifunctional protein involved in autophagy. It can directly bind to LC3 and ubiquitinated proteins, which are ultimately degraded in the lysosome (Bjørkøy et al., 2009). Beclin-1 and ATG5 are key regulatory proteins in autophagosome formation. Feng et al. found that the expression level of p62 considerably increased when autophagy was inhibited (Feng et al., 2017). Qiu et al. (2023) established a mouse poisoning model of Cd and PSNP and observed that combined exposure to Cd and PSNP substantially increased the expression levels of Beclin-1, ATG5, and LC3 but decreased that of P62. Consistent with the abovementioned results, we observed that Cd treatment activated autophagy in the chickens kidney by upregulating and downregulating the expression levels of LC3 and P62, respectively. Conversely, the CdCl_2_ + Nar co-treatment group showed the reduced expression level of LC3 and elevated P62 level, which mitigated autophagy. Nar reduced the CCl_4_-induced elevation of LC3 in rat liver (Ustuner et al., 2020). This result provides support to our research.

## 5 Conclusion

In conclusion, a model of Cd poisoning in chickens was created, and Nar was added to antagonize Cd toxicity. It was found that exposure to CdCl_2_ resulted in decreased body weight and impaired kidney function, as well as induced the apoptosis of renal cells, OS, ER stress, and autophagy in chicken kidneys. However, Nar can effectively combat the adverse effects of CdCl_2_, relieve the CdCl_2_-induced weight loss, restore kidney function, reduce OS and apoptosis, alleviate ER stress, and regulate autophagy. Furthermore, this study provides a valuable foundation for the application of Nar in CdCl_2_ poisoning. However, it is still unclear how Nar regulates ER stress and autophagy induced by CdCl_2_, and its mechanism needs further study.

## Data Availability

The datasets presented in this study can be found in online repositories. The names of the repository/repositories and accession number(s) can be found in the article/Supplementary Material.
